# Unusual occurrence of a DAG motif in the Ipomovirus *Cassava brown streak virus* and implications for its vector transmission

**DOI:** 10.1371/journal.pone.0187883

**Published:** 2017-11-20

**Authors:** Elijah Ateka, Titus Alicai, Joseph Ndunguru, Fred Tairo, Peter Sseruwagi, Samuel Kiarie, Timothy Makori, Monica A. Kehoe, Laura M. Boykin

**Affiliations:** 1 Department of Horticulture, Jomo Kenyatta University of Agriculture and Technology (JKUAT), Nairobi, Kenya; 2 National Crops Resources Research Institute (NaCRRI), Kampala, Uganda; 3 Mikocheni Agricultural Research Institute (MARI), Dar es Salaam, Tanzania; 4 Department of Primary Industries and Regional Development, DPIRD Diagnostic Laboratory Services, South Perth, WA, Australia; 5 School of Molecular Sciences, University of Western Australia, Crawley, Perth, WA, Australia; Washington State University, UNITED STATES

## Abstract

Cassava is the main staple food for over 800 million people globally. Its production in eastern Africa is being constrained by two devastating *Ipomoviruses* that cause cassava brown streak disease (CBSD); *Cassava brown streak virus* (CBSV) and *Ugandan cassava brown streak virus* (UCBSV), with up to 100% yield loss for smallholder farmers in the region. To date, vector studies have not resulted in reproducible and highly efficient transmission of CBSV and UCBSV. Most virus transmission studies have used *Bemisia tabaci* (whitefly), but a maximum of 41% U/CBSV transmission efficiency has been documented for this vector. With the advent of next generation sequencing, researchers are generating whole genome sequences for both CBSV and UCBSV from throughout eastern Africa. Our initial goal for this study was to characterize U/CBSV whole genomes from CBSD symptomatic cassava plants sampled in Kenya. We have generated 8 new whole genomes (3 CBSV and 5 UCBSV) from Kenya, and in the process of analyzing these genomes together with 26 previously published sequences, we uncovered the aphid transmission associated DAG motif within coat protein genes of all CBSV whole genomes at amino acid positions 52–54, but not in UCBSV. Upon further investigation, the DAG motif was also found at the same positions in two other Ipomoviruses: *Squash vein yellowing virus* (SqVYV), *Coccinia mottle virus* (CocMoV). Until this study, the highly-conserved DAG motif, which is associated with aphid transmission was only noticed once, in SqVYV but discounted as being of minimal importance. This study represents the first comprehensive look at *Ipomovirus* genomes to determine the extent of DAG motif presence and significance for vector relations. The presence of this motif suggests that aphids could potentially be a vector of CBSV, SqVYV and CocMov. Further transmission and ipomoviral protein evolutionary studies are needed to confirm this hypothesis.

## Introduction

### Vector transmission associated motifs in *Potyviridae*

Most plant-infecting viruses are transmitted to their host plants by vectors. This interaction between vector and virus varies in duration and specificity, but some common themes have emerged. Firstly, plant viruses encode proteins on the surface of the virion that are essential for transmission and in some cases additional non-structural helper proteins act to bridge the virion to the vector binding site. For example, current evidence suggests that successful transmission of Potyviruses by their aphid vectors depends upon the interaction of two viral proteins; the coat protein (CP) and helper component proteinase–HC-Pro [1, 2]. Potyvirus HC-Pro mediates aphid transmission through protein-protein interactions, serving as a bridge between the coat protein of virions and surfaces of the aphid maxillary food canal and foregut [3, 4]. The most plausible explanation for HC-Pro requirement in potyvirus vector transmission is that it acts as a “bridge” between aphid stylet and virus particle [5]. The bridge hypothesis requires that the helper interact with both the virions and the aphid mouthparts. The actual transmission requires acquisition of viral non-structural HC protein by aphids. Two motifs; KITC and PTK, are highly conserved at the N- and C-terminus of HC-pros, respectively. It has been hypothesized that to bridge virions to aphid alimentary tract, the HC pro acts as a dimer, KITC motif interacts with the aphid stylet and there is PTK to DAG interaction [6, 7].

For viruses lacking HC-Pro or DAG as in the case of *Cucumber mosaic virus*, distinct CP motifs are responsible for transmission by different aphid species. The amino acids in position 25, 129, 162, 168, and 214 of CMV CP are critical for efficient transmission by *Myzus persicae*. In contrast, amino acids in position 129, 162, and 168 are critical for efficient transmission by *Aphis gossypii* [8]. Structural analyses show that amino acids in position 129 and 162 are exposed on virion surface and buried in the folded CP, respectively [9]. The CP is the single structural protein in potyviruses and apart from viral genome encapsidation, it has roles in virus transmission by aphids, cell-to-cell movement and spread [1–3, 10]. A highly-conserved motif of three amino acids Asp-Ala-Gly (DAG), located in the N-terminus of the potyvirus CP is associated with aphid transmission [1, 3]. Using site-directed mutagenesis, it has been demonstrated that certain substitutions in the DAG region result in loss or drastic reduction in aphid transmissibility of *Tobacco vein mottling virus* (TVMV) [1, 2]. However, not all potyviruses with this motif are aphid transmissible and certain modifications of the DAG are also tolerated [11–13]. Until this study, the highly-conserved DAG motif has not been described as a feature in the genomes of viruses in the genus *Ipomovirus* and was unreported for whitefly-transmitted viruses [14]. Similarly, work to transmit CBSD causal viruses with aphids (*Myzus persicae*) or the whitefly *Bemisia tabaci* were either unsuccessful or achieved low transmission rates.

### Spread of major cassava viruses in Africa

Cassava (*Manihot esculanta Cranz*) is a major staple food and income generation crop in eastern Africa [15]. In Kenya, annual cassava production is 1,112,420 tones (FAO, 2013), with the Coast, Western, Nyanza and Eastern regions as the main production regions. Two viral diseases, cassava mosaic disease (CMD) and cassava brown streak disease (CBSD), are the most significant constraints to cassava production in Africa. Seven ssDNA cassava mosaic begomoviruses (family Geminiviridae) have been reported to cause CMD in Africa. In contrast, CBSD is caused by two positive-sense single strand RNA (+ssRNA) viruses; *Cassava brown streak virus* (CBSV) and *Ugandan Cassava brown streak virus* (UCBSV), both belonging to the genus *Ipomovirus*, family *Potyviridae* [16–18], and often jointly referred to as CBSVs. Yield losses due to CBSD in four eastern Africa countries (Kenya, Tanzania, Uganda, and Malawi) were recently estimated at US$750 million annually [19]. Symptoms of CBSD include brown lesions on the stem, veinal and general leaf chlorosis that may result in complete shoot die back with severe disease conditions on above ground tissues. In the starchy storage roots, symptoms appear as root surface fissures, constrictions and corky brown necrotic lesions that are largely responsible for yield losses of up to 70% in individual cassava fields [20–22]. Damage by CBSD not only makes the tuberous roots store poorly but also renders them unsuitable for human consumption or sale in the markets [23]. The losses to CBSD are not only limited to food insecurity in eastern Africa countries, they also undermine efforts spent on breeding for CMD resistant varieties as they are now affected by CBSD.

For several decades, CBSD was thought to be restricted to the coastal lowlands of Kenya, Tanzania, Malawi and Mozambique [24], but it has recently been confirmed at higher altitude areas (greater than 1000 meters) in eastern and central Africa [25–27]. During the last decade, a CBSD epidemic has spread to other inland countries away from coastal East Africa such as Uganda, Rwanda, Burundi, Congo, DR Congo and South Sudan [16, 28–30]. The disease is spread through use of infected stem cuttings as planting material and transmitted semi-persistently by the cassava whitefly *Bemisia tabaci* (Gennadius) (Hemiptera: Aleyrodidae) and spiraling whitefly (*Aleurodicus dispersus*) [31, 32]. However, these studies reported that experimental transmission of CBSV by *B*. *tabaci* takes place with low efficiency [31, 32] or was sometimes unsuccessful [35], so as to be definitively associated with the observed rapid within and between field CBSD spread or the escalating regional epidemic [33]. This has led to suggestions that, besides spread of CBSD either through CBSVs-infected stem cuttings used as planting material or transmission by *B*. *tabaci*, there may be another vector involved in the epidemiology of these diseases.

### Transmission of cassava brown streak viruses

Cassava brown streak disease is spread by movement of infected stem cuttings, but the vector(s) of its causal viruses have remained elusive for decades. Early studies hypothesized several potential vectors including *B*. *tabaci*, *B*. *afer*, *Aleurodicus dispersus*, beetles and various aphid species [31, 32, 34–36]. With very low efficiency, CBSVs have been shown to be transmitted by *B*. *tabaci* and *A*. *dispersus*, 22% and 25.9%, respectively [31, 32]. Unlike cassava mosaic begomoviruses, which are transmitted by *B*. *tabaci* in a persistent manner, CBSVs are transmitted semi-persistently like other *ipomoviruses*, and are not retained for more than 24 h [14, 37]. Once *B*. *tabaci* was identified as a possible vector of the viruses causing CBSD, further transmission studies with other vectors were rarely conducted. Many studies [33, 38, 39] have found that high CBSD incidence in the field is correlated with high whitefly density, which has restricted research on vectors of CBSVs to whiteflies. However, other vectors of plant viruses are observed in cassava fields and surrounding areas but no data exists on these as potential vectors of CBSVs.

### Gene functions in *Potyviridae*

In the *Potyviridae*, the P1 gene has been associated with genome amplification, cell-to-cell movement, and generally virus-host interaction [40–42]. The P1 protein has also been linked to symptom development and host range determination [43]. The P3 is associated with inclusion bodies suggesting that it is involved in replication [44]. The 6K1 and 6K2 are believed to be involved in virus replication. 6K1 is required for viral replication and is an important viral element of the viral replication complex at the early infection stage whereas 6K2, one of the two smallest potyviral proteins, is an integral membrane protein and induces the endoplasmic reticulum (ER)-originated replication vesicles that target the chloroplast for robust viral replication [45]. The CI protein with a conserved RNA helicase domain is also involved in genome replication [40]. The NIa is composed of two parts, one is the VPg and the other is the proteinase [46–48]. The VPg has been implicated in host genotype-specific long distance movement [49] whereas the NIb is thought to be the RNA replicase (RNA dependent RNA polymerase) responsible for virus multiplication. The function of the coat protein is described above.

### Genome organization of cassava brown streak viruses

The complete genome of CBSV was first sequenced in 2009 [50] and to date there are 26 publicly available, and an additional 8 new genomes from this study. Genetic diversity is greater among isolates of CBSV (79.3–95.5% at nucleotide level) than UCBSV (86.3–99.3%) [51, 52]. Studies have shown CBSV to be the more virulent species [22, 53]. Tolerant varieties were infected only with CBSV, but were free of UCBSV, indicating their resistance to the latter, which is a clear distinction between the two viruses. Compared with UCBSV, CBSV isolates have been reported to be more detectable in molecular based assays and possess higher infection rates by graft inoculation and induce more severe symptoms [22]. Although there are currently two species officially recognized by the ICTV, there is strong evidence to support the case for more species [17, 20].

The genomic organization of CBSVs is composed of 10 segments, total size approximately 8.9 to 10.8 kb, coding for a polypeptide with about 2,900 amino acid residues [18, 50, 54]. The CBSVs genome is single stranded and positive sense RNA. The encoded large polyprotein is proteolytically processed into ten mature functional proteins. The genes encoded include (from 5’ to 3’ end) the P1 protein (a serine proteinase), the P3, 6K1, cylindrical inclusion protein (CI), 6K2, Vpg, the nuclear inclusion protein (NIa), the nuclear inclusion protein (NIb), HAM1h and the CP. The genomes also have non-coding regions at the 5’ and 3 ‘termini [50]. The CBSVs lack HC-Pro, instead their P1 serine proteinase down regulates RNA silencing greatly [50]. Absence of the HC-pro in CBSVs is no exception as this occurrence is exhibited in other species of *Ipomovirus* such as *Squash vein yellowing virus* (SqVYV) and *Cucumber vein yellowing virus* (CVYV) in the *Potyviridae* family [55, 56].

We set out to characterize diversity of CBSVs whole genomes from Kenya and, remarkably, discovered an aphid transmission associated motif (DAG) occurrence within the coat protein of CBSV, but not in UCBSV, for all published complete and partial genomic sequences. Therefore, we; (1) investigated the presence or absence of the DAG motif in other *Ipomovirus* whole genome sequences, and (2) characterized 8 new CBSVs whole genomes from Kenya. The presence of the DAG motif in CBSV calls into question our current understanding of vector transmission of these devastating viruses.

## Materials and methods

### U/CBSV isolates sampling sites in Kenya

A survey of farmers’ cassava fields was carried out in four regions of Kenya, namely, Nyanza, Coast, Western and Eastern, between May and October 2013. All farmers gave permission to collect cassava leaf samples from their farms. Selection of cassava fields for sampling was done at regular intervals of 10 Kilometers along major roads [24, 57]. The crops were 6–12 months old, a growth stage of cassava when CBSD above-ground symptoms are clearly visible [24]. During sampling, cassava plants were selected along an X-shaped transect [57] and shoot symptom severity scored on a modified 1 to 5 scale [21]. CBSD shoot symptom severity scale of 1–5 scale represents; 1 = no visible symptoms, 2 = mild vein yellowing or chlorotic blotches on some leaves, 3 = pronounced/extensive vein yellowing or chlorotic blotches on leaves, but no lesions or streaks on stems, 4 = pronounced/extensive vein yellowing or chlorotic blotches on leaves and mild lesions or streaks on stems, 5 = pronounced/extensive vein yellowing or chlorotic blotches on leaves and severe lesions or streaks on stems, defoliation and dieback. Eight (8) CBSD symptomatic leaf samples were collected for CBSVs detection and next generation sequencing (Table 1).

**Table 1:** Cassava brown streak disease samples from Kenya and RT-PCR results.

Sample ID	Region	Location	Geo- coordinates	Field ID	Variety	Infection-PCR results
K01	Western	Bumula	N00.59218, E034.49305	F6S1	Local	CBSV + UCBSV
K02	Western	Bumula	N00.399721, E034.44045	F7S3	Magana	CBSV
K04	Western	Teso	N00.55531, E034.16126	F14S3	Magana	UCBSV
K05	Western	Busia	N00.55531, E034.16126	F14S4	Magana	UCBSV + CBSV
K12	Coast	Malindi	S04.63214, E039.19356	F12S1	Local	UCBSV + EACMV—Ke
K13	Coast	Msambweni	S04.65914, E039.20179	F13S1	Kibandameno	UCBSV + CBSV -Kilifi (Kenya)
K14	Coast	Lungalunga	S04.52837, E039.13629	F14S3	Kibandameno	CBSV + UCBSV, EACMV—Ke,
K15	Coast	Lungalunga	S04.52826, E039.13598	F15S2	Kibandameno	CBSV

### RNA extraction and detection of CBSVs

The CTAB method [58] was used to extract total nucleic acids. Cassava leaf tissue (0.4 g) was weighed and ground in 3 ml of extraction buffer (20 g CTAB 100 ml 1M Tris-HCl pH 8.0, 100 ml 0.5M EDTA pH 8.0, 81.76 g Sodium Chloride, 10 g Sodium Sulphite and 20 g Polyvinylpyrolidone-40 dissolved to make up one litre) in a mortar and pestle with the aid of acid washed sand. The slurry was then transferred to 1.5 ml microfuge tubes and incubated at 65°C for 20 minutes with mixing at 5-minute intervals. Centrifugation was carried out at 13000 rpm for ten minutes before transferring 750 μl of supernatant to a fresh tube. About 750 μl of chloroform: isoamylalcohol (24:1) was added to the tubes. The tubes were well shaken and centrifuged at 13000 rpm for ten minutes. About 600 μl of the aqueous supernatant phase was transferred to a fresh tube and mixed with an equal volume of Chloroform: Isoamylalcohol (24:1). This step was repeated. Aqueous supernatant phase (450 μl) was added to a fresh tube while avoiding the interphase. Nucleic acids were precipitated using ice cold isopropanol (-20°C) before centrifuging at 6500 rpm for ten minutes. Isopropanol was decanted carefully leaving the DNA pellet at the bottom of the tube. The pellet was washed with 500 μl of 70% ethanol before air-drying. The pellets were dissolved in 100 μl sterile distilled water. The isolated total nucleic acids samples were then stored at -80°C until used. 50ng of genomic RNA was used for cDNA synthesis using the RevertAid First Strand cDNA Synthesis Kit (Thermo Scientific™) in a one step process. The resultant cDNA was used as template for setting up PCR using MyTaq™ DNA Polymerase (Bioline™) in a final reaction volume of 25 μl and annealing temperature of 52°C. A universal primer pair for detection of both CBSV and UCBSV in one reaction was used with an expected product size of 344bp and 438-440bp, respectively [59]. The PCR reaction mix consisted of 16.0 μl nuclease free water, 2.5 μl PCR buffer, 2.5 μl MgCl_2_ (2.5mM), 0.5 μl dNTPs (10 mM), 1.0 μl of each primer (10mM) [forward CBSDDF2 5’GCTMGAAATGCYGGRTAYACAA3’ and reverse CBSDDR 5’G[GATATGGAGAAAGRKCTCCI3’], 0.15 μl Taq DNA polymerase and 1.0 μl of cDNA. The PCR conditions were: 94°C for2 min followed by 35cycles of 94°C (30s), 51°C (30s) and 72°C (30s) for denaturation, annealing and extension, respectively. PCR products were analysed by gel electrophoresis in a x1 TAE buffer on a 1.5% agarose gel, stained with ethidium bromide, visualized under UV light and gel picture documented.

### Next generation sequencing

Eight (8) samples were transported to the laboratory and extracted as detailed above. Total RNA was blotted on to FTA cards and later extracted using methods previously described [17]. Total RNA from each sample was sent to the Australian Genome Research Facility (AGRF) for library preparation and barcoding before 100 bp, paired-end sequencing on an Illumina HiSeq2000.

### De novo sequence assembly and mapping

For each sample, reads were first trimmed using CLC Genomics Workbench 7.5 (CLCGW) with the quality scores limit set to 0.01, maximum number of ambiguities to two and removing any reads with <30 nucleotides (nt). Contigs were assembled using the *de novo* assembly function of CLCGW with automatic word size, automatic bubble size, minimum contig length 500, mismatch cost two, insertion cost three, deletion cost three, length fraction 0.5 and similarity fraction 0.9. Contigs were sorted by length and the longest subjected to a BLAST search (blastn and blastx). In addition, reads were also imported into Geneious 6.1.6 [60] and provided with reference sequences obtained from Genbank (see Table 2). Mapping was performed with minimum overlap 10%, minimum overlap identity 80%, allow gaps 10% and fine-tuning set to iterate up to 10 times. A consensus between the contig of interest from CLCGW and the consensus from mapping in Geneious was created in Geneious by alignment with MAFFT [61]. Open reading frames (ORFs) were predicted and annotations made using Geneious. Finalized sequences were designated as “complete” based on comparison with the reference sequences used in the mapping process, and “coding complete” if some of the 5’ or 3’ UTR was missing but the coding region was intact, and submitted to Genbank with the accession numbers MG387652-MG387659 (Table 2).

**Table 2:** Next generation sequencing data (Illumina HiSeq 100 base pair fragments) from cassava plants showing Cassava Brown Streak Disease symptoms collected in Kenya in 2013.

Sample ID	Accession number	Virus	No. of reads obtained	No. of reads after trimming	Number of contigs produced (CLC)	Contig number of positive CBSV/UCBSV and length of postive contig (CLC)	Average coverage (CLCGW)	Number of reads mapped to contig of interest	Ref seq. used for mapping	Length of consensus sequence from mapping (Geneious)	No. reads mapped to ref. sequence	Average coverage (Geneious)	Final sequence length
**K1**	**MG387659**	*CBSV*	15,839,232	12,846,217	790	11 (6081), 12 (2766)	54,17	637, 93	KR108828	8,876	4,808	55	8,748
**K2**	**MG387657**	*CBSV*	18,638,554	13,569,714	3,117	629 (1942), 893 (1931), 285 (1527), 909 (961),1095 (606)	31,16,34,21,18	638, 328, 543, 208, 121	KR108828	8,979	2,626	30	8,745
**K4**	**MG387653**	*UCBSV*	22,774,184	17,035,094	3,466	241(4677), 331(1822), 390(555), 883(1010)	53,102,32,40	34837, 212, 2703, 11742	FN433933	9,437	10,495	17	8,703
**K5**	**MG387654**	*UCBSV*	22,191,388	18,507,658	766	69(4523), 108(1568), 222(574), 452(628), 481(656)	42,49,18,37,26	1908, 770, 105, 247, 173	FN433933	8,824	2,174	25	8,703
**K12**	**MG387655**	*UCBSV*	19,691,178	13,917,214	884	215(2630), 288(2621), 301(2218), 653(780)	64,34,230,252	1717, 1035, 8041, 3483	FN433933	8992	2,349	27	8,614
**K13**	**MG387652**	*UCBSV*	21,979,350	15,966,663	426	148 (562), 222(668), 422(687), 185(733)	18,11,7,8	105, 73, 48, 61	KR108828	8713	602	7	8,703
**K14**	**MG387656**	*UCBSV*	22,543,084	16,565,329	900	18(907), 24(4776), 66(635), 82(1673)	88,169,118,111	816, 8216, 776, 2103	FN433933	8948	8,753	46	8,822

### Genome alignment and annotation

Twenty-six whole genomes (12 CBSV and 14 UCBSV) were downloaded from GenBank and imported into Geneious [60], and the MAFFT plugin [61] was used to align them with the 8 new whole genome sequences obtained in this study. Nucleotide alignments were translated into protein using the translate align option in Geneious and then visually inspected for quality. Annotations were transferred to the 8 new genomes from the 26 previously published genomes using the live annotation option in Geneious, and manually checked for correctness. *Sweet potato mild mottle virus* (SPMMV) (GenBank reference Z73124), *Eggplant mild leaf mottle virus* (EMLMV) (GenBank HQ840786) and *Tomato mild mottle virus* (ToMMov) (GenBank HE600072) were also analyzed for the presence of a DAG motif in the coat protein.

### Gene tree estimation

Individual gene trees were estimated using MrBayes 3.2.1 [62] and run in parallel on Magnus (Pawsey Supercomputing Centre, Perth, Western Australia) utilizing the BEAGLE library [63]. MrBayes 3.2.1 was run utilizing 4 chains for 30 million generations and trees were sampled every 1000 generations. All runs reached a plateau in likelihood score, which was indicated by the standard deviation of split frequencies (0.0015), and the potential scale reduction factor (PSRF) was close to one, indicating that the MCMC had converged.

### CBSV CP sequences in GenBank

After observing the DAG motif in the CBSV whole genome data, all sequences found in GenBank overlapping the DAG region found in the coat protein were obtained. Megablast implemented in Geneious was carried out using the coat protein sequence for CBSV_TZ_Ser_6_KR108830 as the query sequence and all hits matching the query were downloaded and aligned using MAFFT with the default parameters.

### Structure of the CBSV CP

To determine if the DAG motif of CBSV is exposed to the cytosol both I-TASSER (Iterative Threading ASSEmbly Refinement) [64] and Protein Homology/analogY Recognition Engine V 2.0 (Phyre2) [65] were used to predict the structure of the coat protein for CBSV_TZ_Ser_6_KR108830. I-TASSER and Phyre2 are an online platform for protein structure and function predictions using homology modeling.

## Results

### Next generation sequencing

Eight samples were subjected to sequencing on an Illumina Hiseq2000, which resulted in raw reads ranging from 13,839,232 to 22,613,080 for each sample. Upon trimming for quality using CLCGW, these reads were reduced to 12,846,217 to 16,825,692 (Table 2). After de novo assembly of the reads for each sequence using CLCGW, the number of contigs produced ranged from 426 to 3466. The coverage of the contigs of interest was 7 to 230 times. After mapping to reference genomes in Geneious, the lengths of the consensus sequences were 8,713 to 8,992 with coverage of 7 to 90 times with the number of reads mapped to the reference sequence ranging from 602 to 10,495. Final sequence lengths consisted of a consensus between the de novo contig of interest and the mapped consensus sequence and ranged from 8703 to 8822. All eight samples yielded a sequence each, giving a total of 8 sequences; 3 CBSV and 5 UCBSV. The 8 sequences were submitted to GenBank with the accession numbers (MG387652-MG387659).

### DAG motif in CBSV and three other ipomoviruses

**Fig 1:**
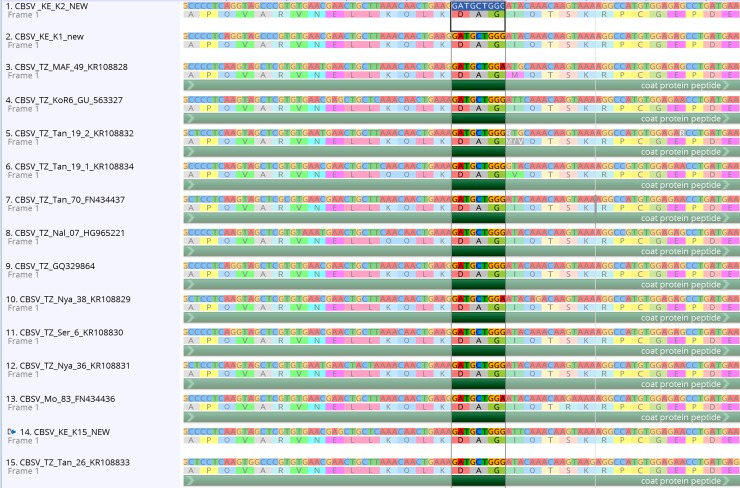
CBSV alignment generated using MAFFT in Geneious. The DAG-amino acid position 52 (highlighted) from the start of the coat protein.

**Fig 2:**
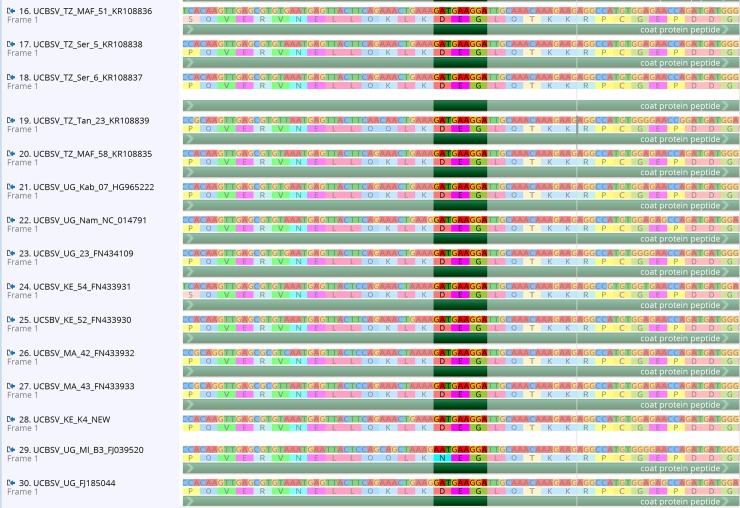
UCBSV alignment generated using MAFFT in Geneious. DEG-amino acid position 52 (highlighted) from the start of the coat protein.

**Fig 3:**
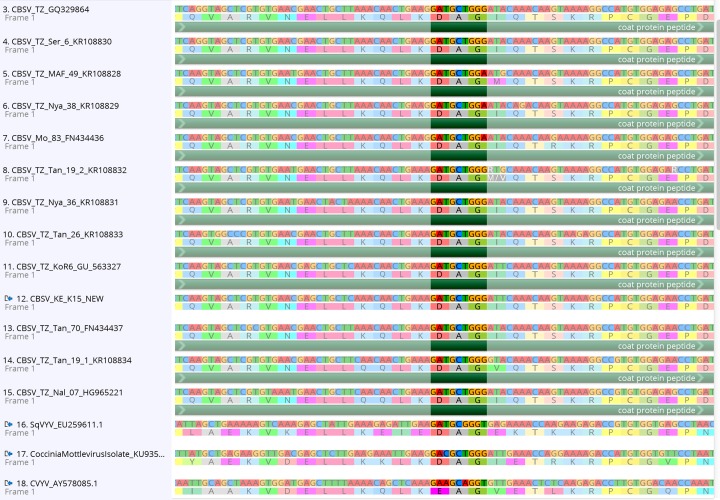
Whole genome sequence alignment of CBSD, SqVYV and CoCMov have the DAG (highlighted), but CVYV does not.

The three (3) new whole genome *Cassava brown streak virus* (CBSV) sequences generated in this study and those recovered from GenBank all have the DAG motif at the N-terminus of the coat protein (13 previously published CBSV whole genomes and an additional 90 coat protein sequences–S1 File). The DAG motif is found at nucleotide position 7768–7776 in the whole genome alignment (Fig 1) and the coat protein position of the DAG motif is 52–54 amino acids from the start of the coat protein. Furthermore, every CBSV sequence from GenBank was downloaded, aligned and all have the DAG motif present (S1 File). In contrast, the *Ugandan cassava brown streak virus* has a DEG motif (15 samples) or NEG (1 sample-FJ039520) at the same location in the alignment (Fig 2). In addition, we found that both SqVYV and CocMov have the DAG motif in the coat protein and CVYV has an EAG at the same position (Fig 3). While the Ipomovirus ToMMov and EMLMV (tentative Ipomovirus) have a DAG motif (but in the NIb), a DAG motif is absent in SPMMV.

### Additional unique CBSV genome features

**Fig 4:**
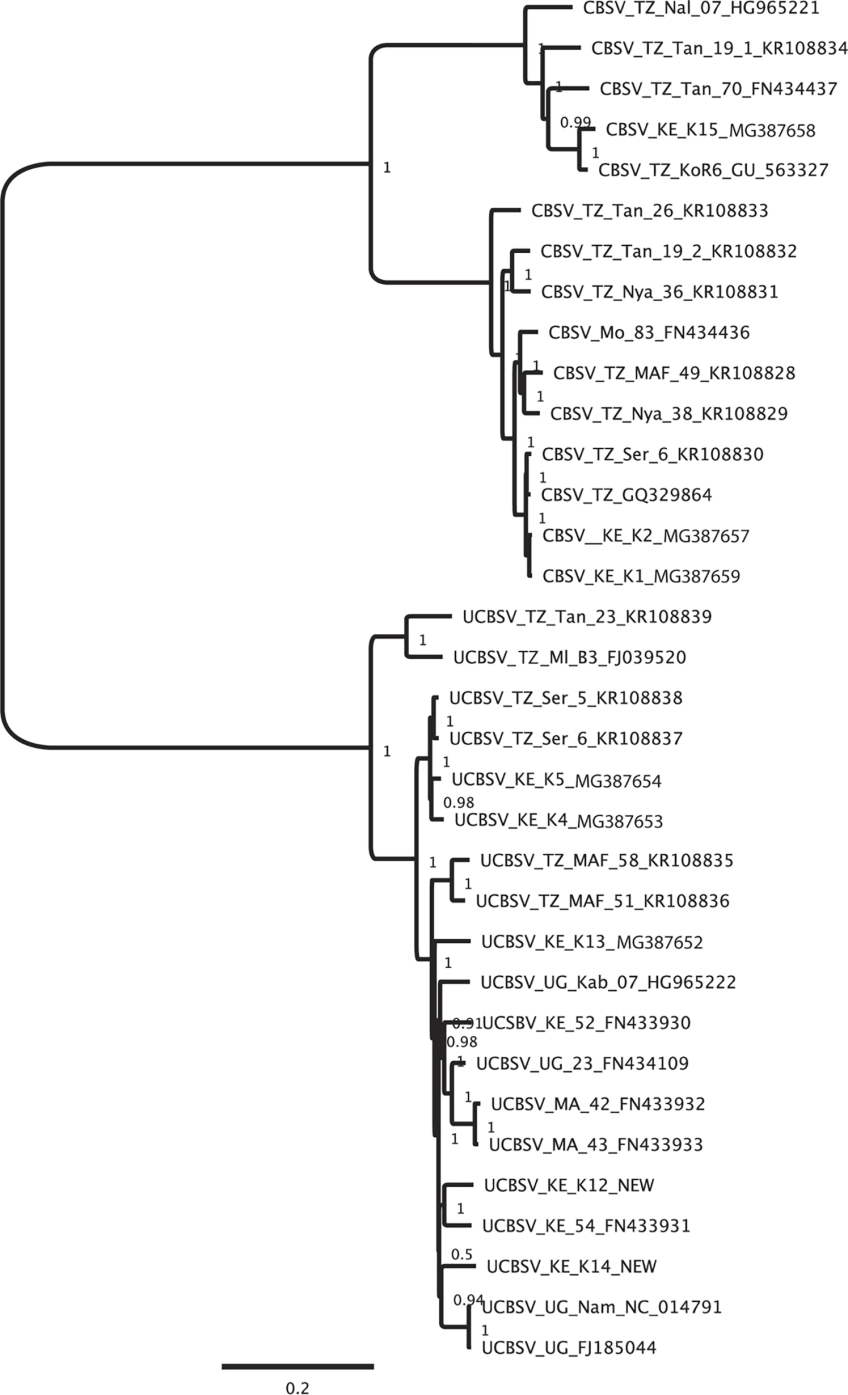
Phylogenetic tree of whole CBSV/UCBSV genomes (nucleotides) following Bayesian analyses using ExaBayes.

The UCBSV genome is longer than that of CBSV and contains other unique characteristics as described here; (i) **Deletion in CBSV P1:** Five CBSV whole genome sequences have a deletion of 4 amino acids in the P1 protein. The sequences with the deletion form a monophyletic group (Fig 4), which contains: CBSV_TZ_07_HG965221, CBSV_TZ_Tan_19_1_KR108834, CBSV_TZ_Tan_70_FN434437, CBSV_KE_K15 (this study) and CBSV_TZ_KoR6_GU563327. This clade corresponds to clade B in Ndunguru *et al*. [17] and was confirmed with a species tree approach in Alicai *et al*. [20]. The UCBSV samples included do not have this deletion. (ii) **PTK and KIC motifs**: CBSV has a PTK in the coat protein (usually found in HC-Pro in other viruses), while UCBSV does not. There is a KIC motif in the CBSV P1, but no KITC motif in either CBSV or UCBSV.

### Phylogenetic relationships of new Kenyan CBSVs whole genome sequences

Samples from Kenya were found in every clade of the CBSVs phylogenetic tree (Fig 4). There are two major clades of CBSV and K15 is found in one clade with 4 other Tanzanian samples. Whole genomes K1 and K2 grouped in the main clade of CBSV forming, their own monophyletic clade and are most closely related to two Tanzanian CBSV isolates CBSV_TZ_GQ329864 and CBSV_TZ_Ser_6_Kr108830. Five new UCBSV whole genomes have been added from Kenya. K4 and K5 form a monophyletic group and are placed next to two Tanzanian UCBSV genomes from Serengeti. While K12 is grouped with another Kenyan whole genome sequence (UCBSV_KE_54_FN433933), K13 and K14 are found on branches by themselves indicating new additions to the genetic diversity of the CBSVs phylogeny.

## Discussion

### Presence of DAG in CBSV, but not UCBSV and their vector transmission

*Cassava brown streak virus* has a DAG motif in the coat protein of all published sequences, including the 3 new whole genomes added in this study, while UCBSV does not. This observation coupled with the low transmission efficiency of CBSV by *B*. *tabaci* [31, 32] strongly suggests a possible alternative vector (or vectors) for CBSV, such as aphids, that requires further investigation. Although Lennon et al. [34] failed to transmit CBSVs with the aphid *Myzus persicae*, three reasons can be used to explain this failure; firstly, it is possible that they worked with UCBSV, which has no DAG, other than CBSV. In 1986, species of CBSVs were not known and therefore it is unknown which virus (CBSV or UCBSV) was used for the transmission. Secondly, low virus titer of CBSVs in the experimental plants may have limited aphid acquisition and subsequent virus transmission. Thirdly, in case their transmission experiments by chance used CBSV which has DAG, it is possible that location or context of the motif was responsible for lack of transmission by aphids. For potyviruses, not only is DAG located near the surface-exposed CP N-terminus and conserved among several isolates transmitted by aphids, but specific context within which it occurs is also of considerable importance [66]. In contrast, *Ugandan cassava brown streak virus* sequences do not have a DAG motif in the coat protein and clearly these data further support observed biological differences between UCBSV and CBSV.

The *Ipomovirus* ToMMoV was initially reported to be experimentally transmitted by aphids [67] and subsequent studies reported it to be inefficiently transmitted by whiteflies[14, 68, 69]. Furthermore, Dombrovsky [69] attributed the higher incidence of ToMMov to high populations of *B*. *tabaci* (MEAM1 or B Biotype) in the field. This scenario is similar to the CBSV and UCBSV hypothesis where higher incidence of the disease symptoms in the field (CBSD) is often attributed to larger populations of the most abundant vector (*B*. *tabaci*) rather than the possibility of the presence of an alternative vector [31, 39].

### Low U/CBSV whitefly transmission rates and difficult reproducibility

Transmission of CBSVs by *B*. *tabaci* and *B*. *afer* has been sporadic and irreproducible. Maruthi [31] reported 22% transmission of CBSV but further review into Table 1 of the paper shows the actual overall transmission rate from controlled glasshouse/insectary experiments in both the United Kingdom and Tanzania reveals 2.9% (7/245 inoculated cassava plants) of the virus. In the same table, specific results for part of the work done in UK where both virus acquisition and inoculation were controlled show transmission rate was 2.3% (5/221 inoculated cassava plants). Bock [35] failed to transmit CBSVs in Kenya with the whitefly *B*. *tabaci*. Furthermore, extremely low transmission rates, inconsistency and irreproducibility in transmission rates were also observed for CBSV [31]. This may in part be due to the semi-persistent transmission, where similar to other *Ipomoviruses*, the CBSVs are not retained in the vector for more than 24 h [14, 37]. In the uncontrolled field studies where CBSD spread was observed, presence of vectors other than whiteflies, including aphids, and their involvement in CBSVs transmission cannot be ruled out. The low transmission and inconsistent results were attributed to technical difficulties in the transmission protocols [31], but it may be that the wrong vector/s or the wrong/multiple viruses species were used. Interestingly, in 2005 it was thought that there was one species of *B*. *tabaci* but we now know there are at least 34 species [70], so it is difficult to tell which species of *B*. *tabaci* was used in the transmission studies. Also, there are now more species of CBSVs than previously thought [17, 20], so the combination of vector and viruses was not precisely known in Maruthi [31].

Furthermore, studies in Malawi show there being no clear correlation between whitefly populations and CBSD, despite widespread occurrence of the disease. All monitoring studies carried out by the Cassava Diagnostics Project (CDP Bill and Melinda Gates Foundation Grant no. 51466) indicate whitefly *B*. *tabaci* populations to be generally low in most cassava-growing areas in Malawi compared to what is reported in East Africa. On average the highest mean whitefly populations were recorded to be 4.8 in 2009/2010, 4.0 in 2011, 15.3 in 2013 and 5.5 in 2015 in country-wide surveys carried out in Malawi (Unpublished data). Additionally, it was confirmed through sequencing of the mitochondrial cytochrome I (mtCOI) gene that the predominant whitefly species in Malawi is *B*. *afer* and not *B*. *tabaci* (Boykin personal communication). In Malawi, the spread of CBSD seems to be due mainly to planting disease affected cassava stem cuttings than vector borne infections. However, there is possibility of involvement of another insect vector (such as aphids) in the spread of CBSD. A report on outreach activities to demonstrate ‘benefits of using clean/disease-free virus indexed planting materials’, indicated a susceptible cultivar Matakolembwende which started with no CBSD, to build up high disease incidence levels when planted in Karonga district, a high CBSD pressure area in northern Malawi (Unpublished Malawi CDP Country report, 2016). In this area, whitefly populations were equally low during the study as elsewhere in the country; leaving the cause of the high rate of spread of CBSD in local susceptible cultivar in great doubt. A recent paper from Kenya also confirmed a lack of correlation between whitefly numbers and CBSD incidence [71]

### How did scientists previously miss the DAG motif in CBSV?

**Fig 5:**
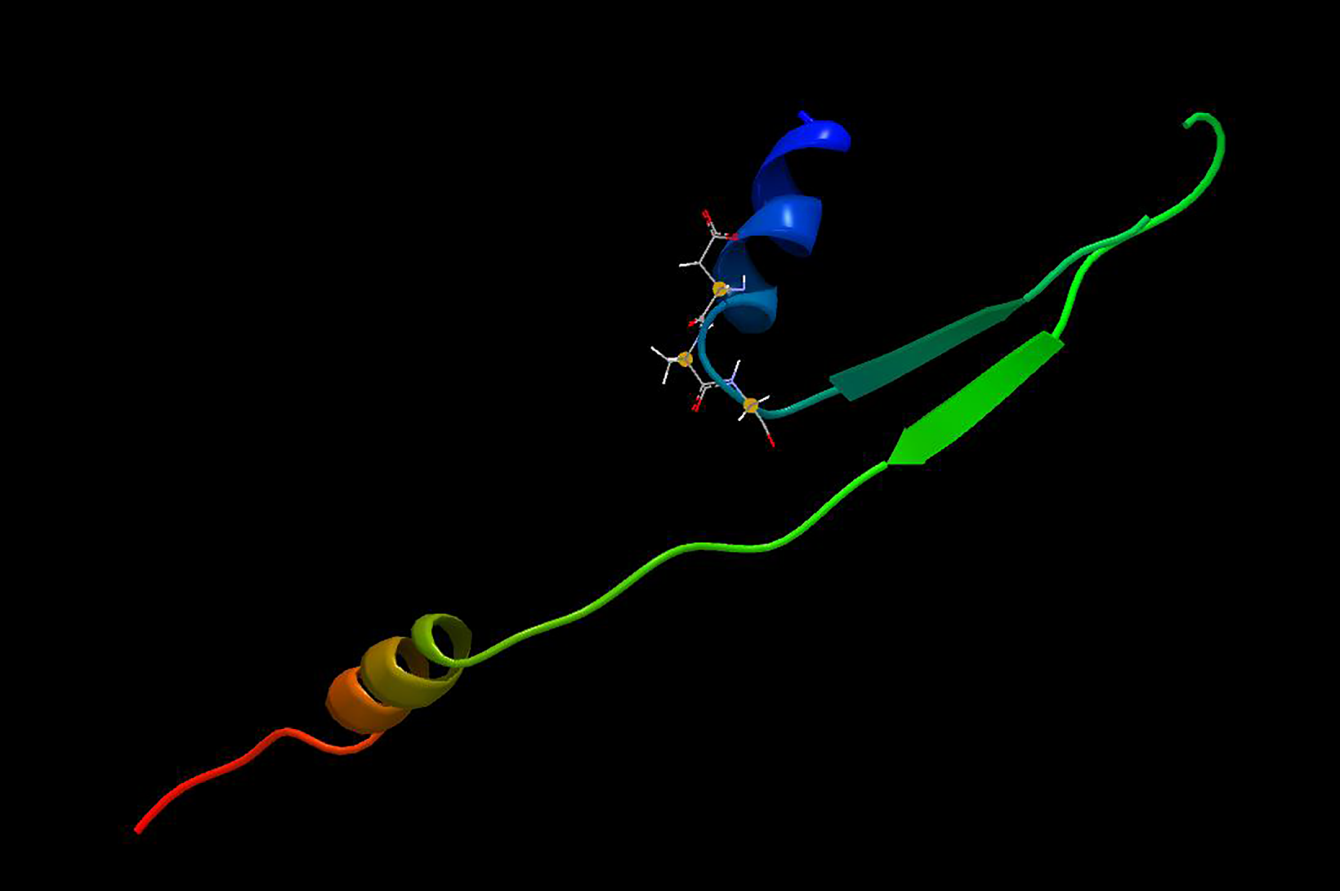
Protein 3D structure of CBSV coat protein generated using Phyre2. The DAG motif is shown with chemical structures.

There are several reasons the DAG motif in CBSV has been missed. Firstly, non-overlapping genomic regions of coat protein have been amplified in most past diagnostics. The DAG motif is 52–54 amino acids from the start of the coat protein and the diagnostic primers start at position 100 and beyond; therefore, people have missed it. Secondly, CBSV DAG could have been missed due to lack of focused scrutiny for it, as all related published literature [2, 14, 72–74] concluded that the DAG motif is not present in the *Ipomoviruses*. Thirdly, several publications have mislabeled sequences as CBSV and yet they are for UCBSV. For example, Dombrovsky [68, 75] has labeled the GenBank accessions FN433930 [75] and ACM48176 [68] as CBSV when they are UCBSV. This mislabeling meant these studies included two UCBSV sequences and no CBSV sequences in the analyses therefore, the DAG motif was never observed. In addition, most scientists may not have translated their nucleotide alignments into amino acids—a very crucial step in understanding coding regions of genomic information. Lastly, one study [72], noticed the DAG motif in SQVYV but thought that it was too far away from the N-terminus to be recognized by aphids, but our study shows it is exposed and accessible to the aphid stylet (Fig 5).

### *Potyvirus* aphid transmission

Non-colonizer aphid species contribute more to potyvirus spread than colonizer species [76, 77]. Studies have shown that aphids making the first probes, coupled with frequent intracellular punctures made a few minutes after landing, transmit potyviruses with high frequency [78, 79]. Ndunguru et al. [80] serologically detected aphid-transmitted sweet potato feathery mottle virus (SPFMV) in 45% of symptomatic sweet potato plants in Tanzania and Uganda. Surprisingly, no aphids were observed in these plants suggesting that transmission of SPFMV may have occurred when aphids briefly fed on them and then moved to their preferred hosts. Other examples of non-colonizing aphids transmitting viruses and causing extensive damage include *Myzus persicae* in *Pisum sativum* (field pea) non-persistently transmitting *Pea seed-borne mosaic virus* (PsbMV). Despite no colonization ever being recorded in field pea, winged aphids are a major driver of virus infection, along with the level of seed infection present at the time of planting [81]. This scenario is not unlike that which we are proposing may occur in East Africa with CBSV—where even a low level of CBSD incidence in a field can provide diseased plants for transient aphids to feed on, and spread virus from as they move through the crop. If such a system was proven, it could have major implications for recommended integrated pest management strategies in the future. As a result, for management of PsbMV in field pea and *Bean yellow mosaic virus* infecting *Lupinus angustifolius*, closure of the crop canopy as soon as possible to reduce number of aphids drawn to land on bare ground have been recommended [81, 82]. Similarly, the recommended row spacing for planting of cassava cuttings may be just one part of the cassava system requiring review.

It is well known in plant virology that continuous mechanical passage of an aphid transmitted plant virus in a greenhouse situation often ends in the virus isolate losing its ability to be transmitted by aphids [83]. In this system we are proposing, the continuous propagation of cassava by stem cuttings undoubtedly provides a constant source and mode of spread for CBSD. Currently, it is too difficult to determine context of the DAG motif in the CBSV genome. We don’t know whether it has been acquired from an ancestral virus or it has always been there. Perhaps the evolutionary pressure for aphid transmissibility to be maintained is not so high as in other viruses where seed transmission is not known to occur.

Seven species of aphids have previously been tested for transmission of CBSVs. Bock [35] tested six species of aphids (*Aphis craccivora*, *A*. *gossypii*, *A*. *nerii*, *Rhopalosiphum maidis*, *R*. *rufiadominalis* and *Schoutedenia lutea*), and Lennon et al. [34] tested *Myzus persicae* with no transmission success. However, they could have been using UCBSV in these transmission studies and not CBSV, because knowledge and distinguishing diagnostics of the two-virus species were not available then. The transmission studies also used the indicator plants *Nicotiana debneyi* or *N*. *tabacum*, and not cassava. There are many species of aphids present in East Africa [84] and several species of CBSV and UCBSV [17, 20]. It is therefore possible that incorrect combinations of vector and virus were the reason these older transmission studies using different aphid species were inconclusive.

Relatedly, in the newly described CoCMov, another *Ipomovirus* which we have shown to contain a DAG motif in its coat protein gene (Fig 3), very low transmission with *B*. *tabaci* MED species was reported [85]. Transmission of CoCMov with the aphid species *Myzus persicae* was not successful, however detailed data was not shown. Inefficient transmission of CoCMov by *B*. *tabaci* was attributed to poor transmissibility or low incidence of the virus source, *Coccinia grandis*. In fact, the low transmissibility could be due to the fact that wrong virus-vector combinations were tested.

The final *Ipomovirus* with a DAG motif in its coat protein is SqVYV, reported to be transmitted by *Bemisia tabaci* [72]. Interestingly, Adkins et al. [69] noticed that the SqVYV CP contained a DAG motif at residues 52 to 54 from its N terminus. Typically, DAG motif is present 5 to 13 residues from the exposed N terminus of a coat protein gene and has been linked to aphid transmission in members of genus Potyvirus [86, 87]. Adkins et al. (2007) determined that SqVYV is not aphid transmissible by *Myzus persicae*. They concluded that lack of SqVYV aphid transmission is perhaps explained by an incorrect sequence context surrounding the DAG motif [13] or its occurrence too far from the N-terminus to be exposed [87]. It is surprising that the presence of DAG motif in *Ipomoviridae* genomes has not received more attention before now. Our modelling of genomes herein suggests that the DAG may be in a position that potentially influences *Ipomovirus* aphid transmission. More work is needed in the future, with multiple aphid species, not just the common ones. Studies that include sticky traps in fields, changed regularly will be required to identify types of transient aphids that pass over a field, and pan traps for capturing those lower in the canopy. As previously reported [88], a helper virus with HCPro can assist transmission of another virus and shown in Adkins et al. [72]. In cassava subsistence cultivation where farmers intercrop cassava with sweet potato, maize and/or cowpeas, there is a possibility that visiting aphids from nearby potyvirus-infected plants can assist the transmission of CBSV into cassava that is worth studying to determine the role of DAG in CBSV and for CBSD. Dombrovsky et al. [14] reported SqVYV is moderately transmitted by *B*. *tabaci* so there is clearly a need to further explore transmission studies with different species of aphids.

### Whole genome phylogenetic analyses of Kenyan U/CBSV isolates

Our new whole genomes from Kenya are found in every clade of the CBSVs phylogenetic tree (Fig 4), indicating no single introduction of these viruses to Kenya. There are two major clades in CBSV and K15- from the Kenyan Coast (Lungalunga) is found in one clade with 4 other Tanzanian isolates. The remaining two CBSV genomes; K1 and K2 are samples from western Kenya (Bumula). These group in the main clade of CBSV, but form their own monophyletic clade and are most closely related to two Tanzanian CBSV isolates; (CBSV_TZ_GQ329864 and CBSV_TZ_Ser_6_Kr108830). Five new UCBSV whole genomes have been added from Kenya. K4 and K5 form a monophyletic group and are placed next to two Tanzanian genomes from Serengeti. While K12 is grouped with another Kenyan whole genome sequence (UCBSV_KE_54_FN433933) K13 and K14 are found on branches by themselves indicating new additions to the genetic diversity of the CBSVs phylogeny.

### Power of advanced software in scientific discovery

Utilizing the most advanced genetic sequencing technology, coupled with high performance computing, and user-friendly alignment visualization tools, we have uncovered patterns in CBSVs genomic data that were previously undiscovered. Translating nucleotides into amino acids and critically analyzing the resulting alignment before proceeding to downstream analyses led us to discover the DAG in CBSV genomes and not UCBSV. The importance of translating nucleotide sequences into amino acids cannot be underestimated as demonstrated here. These observations would not have been possible even ten years ago, and the pace of advancement in software and hardware capabilities ensures that this type of analysis and investigation will become commonplace. This further advances our understanding of how genomics and biological factors interact, and leads us to new routes of investigation in the field, and faster than ever before.

### Future directions

The first actions of further investigation must be an attempt to understand the diversity of aphid species present in and around cassava fields in east Africa. This includes morphological and molecular identifications. Second will be to collect and maintain populations of common species for CBSVs aphid transmission experiments. When this occurs, it must be with isolates of CBSV whose whole genome sequence is known, and an isolate from each proposed new species group needs to be included. This is the only way to answer the most pressing questions. It is becoming clear that there are many as yet undiscovered species within the Ipomoviruses. As the genetic landscape of these viruses increases due to advances in NGS technologies we will gain a clearer picture of the virus species and be able to pin point the most recent common ancestor to these sequences. This will shine a light on the evolutionary history of the presence of the DAG motif in ipomoviruses, and their potential transmission by other vectors like aphids. In addition, a crystal structure of CBSV coat protein is needed to identify exactly where the DAG motif is located. Current predictions are based on homology to tobacco mosaic viruses, which are clearly divergent. There are opportunities to develop better diagnostic tools for both virus species confirmation and vector identification particularly in-field, to ensure researchers know exactly what they are taking back to the laboratory and greenhouse.

## Supporting information

S1 File 111 cassava brown streak virus sequences downloaded from GenBank all with the highly conserved DAG motif in the coat protein. (PDF)
