# Supplementary material for: Unusual occurrence of a DAG motif in the Ipomovirus Cassava brown streak virus and implications for its vector transmission
Source: PLoS One. 2017 Nov 20;12(11):e0187883. doi: 10.1371/journal.pone.0187883 (PMC5695790; doi:10.1371/journal.pone.0187883)
Supplement: S1 File — (PDF) [file pone.0187883.s001.pdf]

Appendix 1. 111 CBSV sequences downloaded from GenBank all with the highly conserved DAG motif in the coat protein.

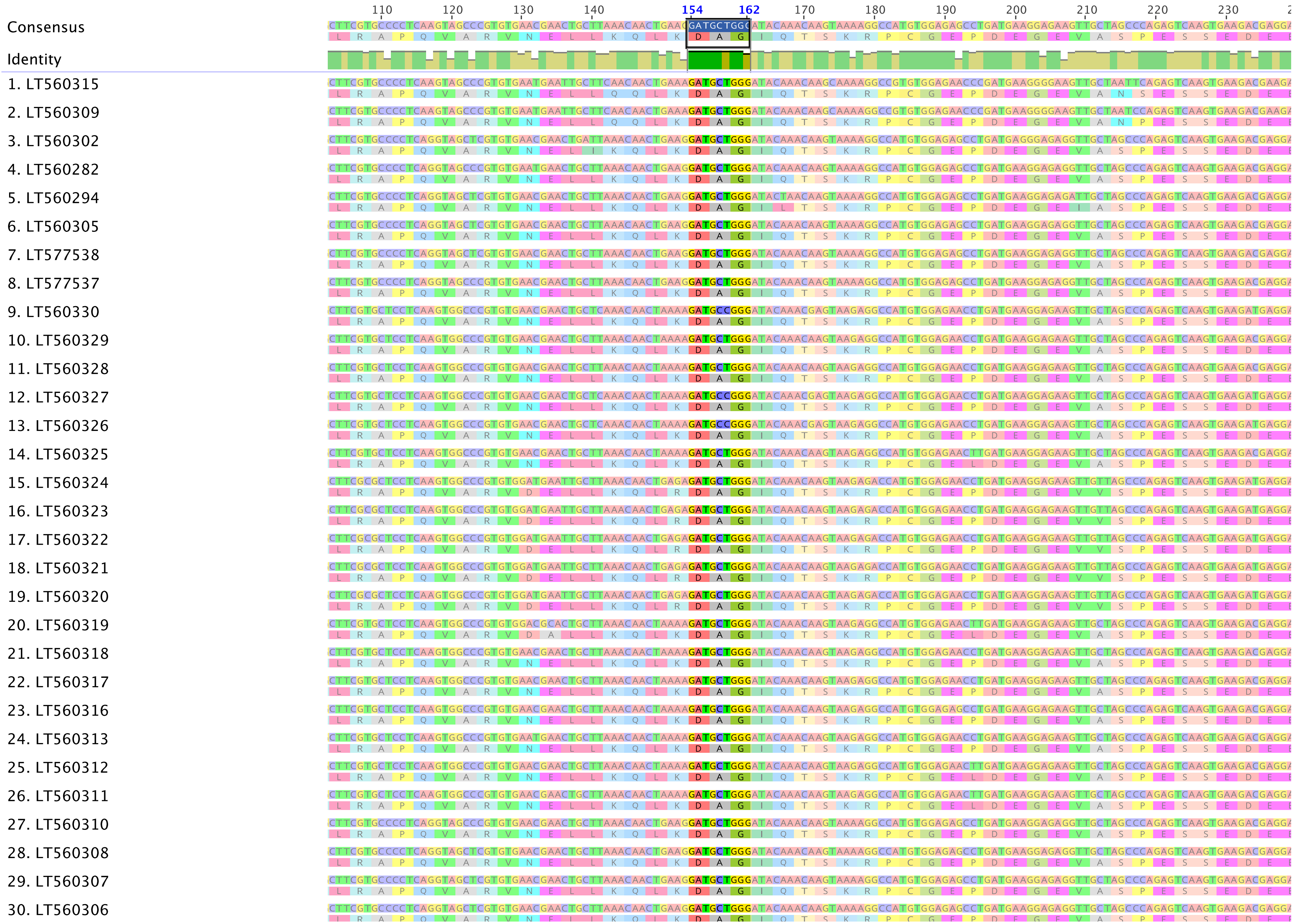

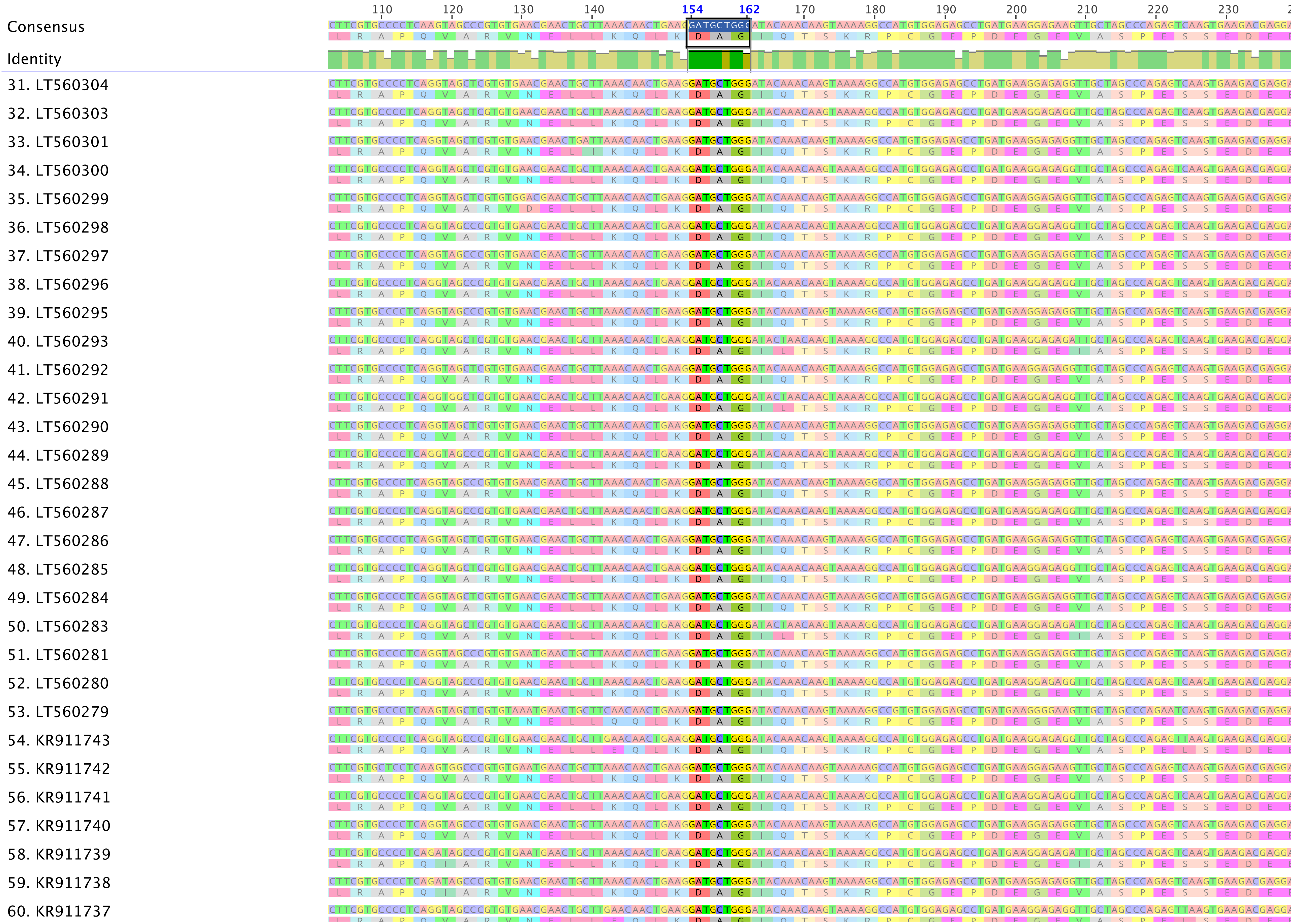
110
120
130
140
154
162
170
180
190
200
210
220
230
Consensus
Identity
31. LT560304
32. LT560303
33. LT560301
34. LT560300
35. LT560299
36. LT560298
37. LT560297
38. LT560296
39. LT560295
40. LT560293
41. LT560292
42. LT560291
43. LT560290
44. LT560289
45. LT560288
46. LT560287
47. LT560286
48. LT560285
49. LT560284
50. LT560283
51. LT560281
52. LT560280
53. LT560279
54. KR911743
55. KR911742
56. KR911741
57. KR911740
58. KR911739
59. KR911738
60. KR911737

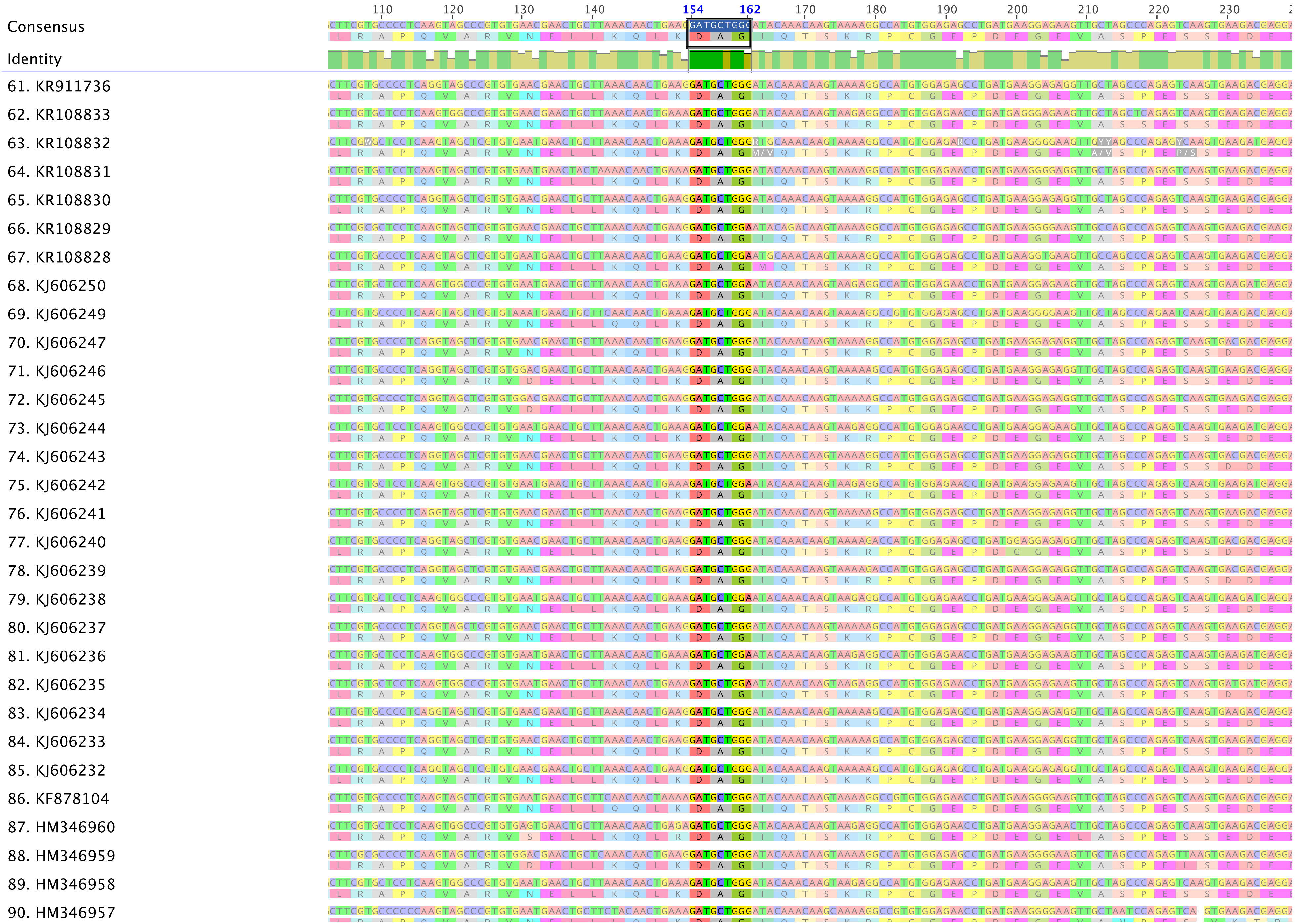

110
120
130
140
154
162
170
180
190
200
210
220
230
Consensus
Identity
61. KR911736
62. KR108833
63. KR108832
64. KR108831
65. KR108830
66. KR108829
67. KR108828
68. KJ606250
69. KJ606249
70. KJ606247
71. KJ606246
72. KJ606245
73. KJ606244
74. KJ606243
75. KJ606242
76. KJ606241
77. KJ606240
78. KJ606239
79. KJ606238
80. KJ606237
81. KJ606236
82. KJ606235
83. KJ606234
84. KJ606233
85. KJ606232
86. KF878104
87. HM346960
88. HM346959
89. HM346958
90. HM346957
